# Influenza virus detection: driving change in public health laboratories in the Western Pacific Region

**DOI:** 10.5365/wpsar.2018.9.5.006

**Published:** 2018-09-05

**Authors:** Raynal C Squires, Patrick C Reading, Sheena G Sullivan, Ian G Barr, Frank Konings

**Affiliations:** a WHO Regional Office for the Western Pacific, Manila, Philippines.; b WHO Collaborating Centre for Reference and Research on Influenza, Peter Doherty Institute for Infection and Immunity, Melbourne, Australia.; c WHO Regional Office for the Eastern Mediterranean, Cairo, Egypt.

As we observe the 100th anniversary of the 1918 influenza pandemic, we are reminded of the importance of preparedness for and adequate response to influenza, and the critical role of influenza surveillance through laboratory detection. Influenza virus detection has helped drive the development of diagnostic and virology laboratories in the World Health Organization (WHO) Western Pacific Region over the last 10–15 years, at the same time strengthening their capacity to detect and respond to infectious threats beyond influenza. Such cross-cutting approaches are advocated under the Asia Pacific Strategy for Emerging Diseases and Public Health Emergencies (APSED III), (*1*) which continues to guide Member States in advancing implementation of the International Health Regulations, 2005 (*2*) and has a dedicated focus on strengthening laboratory capacities.

For over 65 years, worldwide surveillance of influenza has been conducted through the WHO Global Influenza Surveillance and Response System (GISRS) laboratory network. (*3*) National Influenza Centres (NICs, usually national or provincial diagnostic or reference laboratories) report in-country influenza activity to WHO and refer a subset of clinical specimens or virus isolates to WHO collaborating centres (WHO CCs) for detailed antigenic and genetic characterization, antiviral drug susceptibility testing and other analyses. WHO CCs, H5 Reference Laboratories, Essential Regulatory Laboratories and other experts meet twice-yearly to review laboratory and epidemiological data to assist WHO in making recommendations on suitable virus strains for seasonal and pandemic influenza vaccines. (*3*)

**Figure 1:**
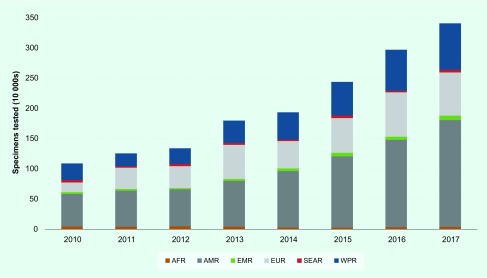
**Number of clinical specimens tested for influenza by the GISRS laboratory network in the six WHO regions from 2010 to 2017**

In 2017, GISRS laboratories in the Western Pacific Region tested nearly 800 000 specimens for influenza (**Fig. 1**). GISRS monitoring of circulating influenza viruses in humans enables timely detection and reporting of significant changes in seasonal influenza viruses such as the emergence of the influenza A(H1N1) pandemic virus in 2009 and the rapid global spread of oseltamivir-resistant seasonal H1N1 viruses in 2007–2008. (*4*) It also increases the speed with which novel influenza A subtypes with pandemic potential can be detected, like avian influenza A(H7N9). Through the Pandemic Influenza Preparedness Framework, vaccine, antiviral and diagnostics manufacturers benefitting from the sharing of viruses and data collected through GISRS return a monetary contribution to WHO to help strengthen surveillance in the laboratory network, particularly in countries with lower capacity. (*3*) The system does have limitations, however, that reflect country capacities and priorities. For instance, the resources needed to maintain NICs and surveillance are primarily concentrated in larger Western Pacific Region Member States rather than small Pacific islands, and countries with unusual numbers of cases are more likely to prioritize sharing. Nevertheless, sharing is key to the success of GISRS, and attention, support and advocacy should be invested into enhancing country participation.

[**insert** Figure 1]

Fast, accurate and reliable methods for the diagnosis of influenza virus infection are needed for surveillance of emerging viruses, outbreak management, early antiviral treatment, prophylaxis and infection control. The traditional method of influenza virus detection by isolation in eggs or cell culture followed by antigenic typing is labour-intensive and time-consuming, particularly in the context of an outbreak. Polymerase chain reaction (PCR) techniques developed in the past 25 years enabled the rapid and specific detection of viral nucleic acid sequences, becoming the gold standard for diagnosis and surveillance. Since 2004, PCR has been instrumental in the early detection of various zoonotic influenza viruses in humans, including A(H5N1), A(H5N6), A(H7N9), A(H9N2) and others in the Western Pacific Region. (*5*) NICs worldwide now routinely perform conventional, real-time and/or multiplex PCR for molecular detection of influenza viruses. In addition to PCR, some NICs in the Western Pacific Region have introduced other molecular tests (e.g. sequencing, pyrosequencing, next-generation sequencing) as well as serological assays (e.g. haemagglutination inhibition, virus neutralization) and testing for sensitivity to antiviral drugs. Nevertheless, serological and drug-sensitivity assays require influenza viruses to be amplified from clinical material, meaning that laboratories performing these tests must still maintain good capacity for traditional methods.

NICs are mandated to maintain high technical capacity for influenza testing (*3*) and are evaluated on the quality of their testing through external quality assessment (EQA). Following several outbreaks of human infection with avian influenza A(H5N1), WHO initiated an EQA programme in 2007 to monitor the quality of PCR detection of influenza virus, and to identify gaps in testing and potential areas of support to NICs. The programme has since grown in sophistication and now includes seasonal influenza A, influenza B and other non-seasonal influenza A viruses responsible for human infections, as well as drug susceptibility analysis. In the Western Pacific Region, the percentage of NICs scoring fully correct results for the detection of influenza virus by PCR increased from 57.1% in 2007 (Frank Konings, WHO, personal communication, 2018) to 84.2% in the 2017 round of the EQA programme. (*6*) In a related first-run EQA to evaluate performance in the isolation and identification of influenza viruses in cell culture, over two-thirds of regional NICs had 80% or more correct results. (*7*)

As the majority of NICs in the Region actually test a broad range of infectious diseases or are housed in institutions that do, the benefits of technical and human resource strengthening through GISRS have been crosscutting. Annual NIC meetings bring together experts to discuss progress, obstacles and best practices, helping to strengthen countries’ laboratory technical capacity through better coordination, a key strategic action in APSED III. Molecular testing available in the GISRS laboratory network has also formed the basis of regional preparedness for detection of emerging pathogens, including Middle East respiratory syndrome coronavirus (*8*) and Zika virus. (*9*) Similarly, drawing on the established EQA programme for PCR detection of influenza virus, WHO worked with WHO CCs to develop and distribute an EQA for arboviruses to the network, starting with dengue virus in 2013 and now including chikungunya, Zika and yellow fever viruses. (*10*) Not solely an evaluation of performance, EQA helps to reveal problems in general laboratory practices, improves the reliability of delivering accurate test results in a timely manner and is usually required for laboratory accreditation. (*11*) Finally, there has long been strong focus on NIC staff development through training in data management and analysis, virus isolation, sequencing and bioinformatics, drug susceptibility testing, infection prevention and control and shipping of infectious substances. These skills are clearly applicable beyond influenza work, multiplying the benefits of the initial investment manyfold.

Since the 1918 pandemic and the later introduction of GISRS, regional NICs have been maintaining traditional methods, incorporating new technologies and building human resource capacity to help strengthen preparedness and response to influenza. The cross-cutting advantages generated and the benefits of sharing and collaboration through GISRS contribute to better preparedness for future outbreaks of influenza and other infectious diseases.
